# Novel cell separation method for molecular analysis of neuron-astrocyte co-cultures

**DOI:** 10.3389/fncel.2014.00012

**Published:** 2014-01-30

**Authors:** Andrea Goudriaan, Nutabi Camargo, Karen E. Carney, Stéphane H. R. Oliet, August B. Smit, Mark H. G. Verheijen

**Affiliations:** ^1^Department of Molecular and Cellular Neurobiology, Center for Neurogenomics and Cognitive Research, Neuroscience Campus Amsterdam, VU University AmsterdamAmsterdam, Netherlands; ^2^INSERM U862, Neurocentre MagendieBordeaux, France; ^3^Université de BordeauxBordeaux, France

**Keywords:** method, co-culture, astrocytes, transcriptional activation, isolation approaches, neuron-glia interaction

## Abstract

Over the last decade, the importance of astrocyte-neuron communication in neuronal development and synaptic plasticity has become increasingly clear. Since neuron-astrocyte interactions represent highly dynamic and reciprocal processes, we hypothesized that many astrocyte genes may be regulated as a consequence of their interactions with maturing neurons. In order to identify such neuron-responsive astrocyte genes *in vitro*, we sought to establish an expedited technique for separation of neurons from co-cultured astrocytes. Our newly established method makes use of cold jet, which exploits different adhesion characteristics of subpopulations of cells (Jirsova etal., 1997), and is rapid, performed under ice-cold conditions and avoids protease-mediated isolation of astrocytes or time-consuming centrifugation, yielding intact astrocyte mRNA with approximately 90% of neuronal RNA removed. Using this purification method, we executed genome-wide profiling in which RNA derived from astrocyte-only cultures was compared with astrocyte RNA derived from differentiating neuron-astrocyte co-cultures. Data analysis determined that many astrocytic mRNAs and biological processes are regulated by neuronal interaction. Our results validate the cold jet as an efficient method to separate astrocytes from neurons in co-culture, and reveals that neurons induce robust gene-expression changes in co-cultured astrocytes.

## INTRODUCTION

During central nervous system (CNS) development, glia and neurons are targeted to their proper location in the brain and the appropriate contacts between neurons, as well as neurons and glia cells, are established. While neuroscience research historically has focused on synaptic contacts between neurons, the last couple of decades it has become apparent that reciprocal neuron-glia interactions are crucial for correct brain functioning. The functionally diverse glia-neuron interactions rely on a range of both contact-dependent mechanisms as well as soluble factors (Eroglu and Barres, 2010; Giaume et al., 2010). The development of elaborate *in vitro* systems, using highly purified cell populations, has strongly contributed to the elucidation of the molecular effects of glia on neuronal development and function (Ullian et al., 2001; Thomson et al., 2008; Jarjour et al., 2012). Specifically, the use of astrocyte-neuron co-cultures has led to the identification of several astrocyte-derived signals that induce neuritogenesis and synaptogenesis, at least *in vitro*. For instance, lipids are secreted by astrocytes in high quantities and are well known to promote neurite outgrowth and synapse formation, (Mauch et al., 2001; Medina and Tabernero, 2002; Goritz et al., 2005; Camargo et al., 2009; Verheijen et al., 2009). Other astrocyte-secreted factors that were shown to influence synapse development are extracellular matrix molecules, such as thrombospondins and glypicans (Clarke and Barres, 2013).

Whereas new astrocyte factors affecting neuronal function are likely to be discovered, a prominent deficit in our knowledge is how expression and secretion of these astrocyte factors are controlled. Neuronal synaptic activity has been shown to induce transcriptional activity in astrocytes (Mendez et al., 2004). It thus follows that synaptic activity and neuron-glia communication will likely elicit gene expression changes in glia, which in turn might reciprocally affect the process of synapse maturation and synaptic transmission. In order to identify neuron-responsive astrocyte genes, we established a developmental *in vitro* transcriptional profiling experiment using neuron-astrocyte co-cultures. To specifically isolate astrocytes from the co-culture, we successfully developed a fast method, named “cold jet” that exploits different adhesion characteristics of subpopulations of cells (Jirsova et al., 1997). The method lacks disadvantages of other methods, e.g., that of fluorescence activated cell sorting (FACS) or magnetic bead-based cell sorting involving protease-mediated dissociation of cells (Lovatt et al., 2007; Foo, 2013), which cannot be efficiently conducted at low temperatures, thereby compromising both protein and mRNA quality. Using cold jet-mediated isolation of astrocytes, followed by microarray analysis, we identified nearly 400 mRNAs significantly regulated in astrocytes when co-cultured with neurons. The resulting database confirms the validity of the used cold jet method for separation of astrocytes and neurons, and represents an important gene source toward understanding of neuron-glia communication.

## METHODS

### CELL CULTURE

Neuron-astrocyte co-cultures were done using rat astrocytes, because of tissue culture advantages of rat over mouse astrocytes, and mouse neurons to make it possible for future experiments to use neurons of genetically modified mice. Importantly, in these co-cultures, rat astrocyte provide the required support for optimal synaptic functioning of co-cultured mouse neurons (Wierda et al., 2007), an important requirement for our screen of neuron-induced expression changes in astrocytes. Primary rat astrocytes were obtained from Wistar rat pups (P1). Briefly, dissected cortices were treated with trypsin (Gibco) followed by addition of trypsin inhibitor and gentle trituration, after which cells were resuspended in Dulbecco’s modified Eagle’s medium (DMEM) + Glutamax (Gibco) containing 10% fetal bovine serum and cultured in non-coated plastic flasks. Medium was changed every 2 days. The purity of astrocyte cultures was determined by immunostaining for glial fibrillary acidic protein (GFAP) and S100β, while counterstained for all cell nuclei using Hoechst, and was determined to be higher than 98%. For neuron-astrocyte co-cultures, astrocytes were plated at a density of 30K/well in 12-wells plates in DMEM + 10% medium, which resulted in an about 80% confluent monolayer at day *in vitro* (DIV) 3, after which neurons were plated. For co-plating of neurons, hippocampi of E18 mouse embryos were collected in Hanks buffered salts solution (HBSS; Sigma), buffered with 7 mM 4-(2-hydroxyethyl)-1-piperazineethanesulfonic acid (HEPES). After removal of the meninges, hippocampi were minced and incubated for 20 min in 0.25% trypsin in HBSS at 37°C. After washing, the neurons were triturated with fire-polished Pasteur pipettes, counted, and plated in culture medium [Neurobasal medium (Invitrogen, Carlsbad, USA) supplemented with 2% B-27 (Invitrogen), 1.8% HEPES, 1% glutamax (Invitrogen), 1% Pen/Strep (Invitrogen), and 0.2% beta-mercaptoethanol] at a density of 60K/well onto the astrocyte monolayers in 12-well plates (4 cm × 4 cm; Greiner bio-one; Wierda et al., 2007). Wells were coated with 20% d-poly-lysine (Sigma) and 20% collagen diluted in a 17 μM acetic acid H_2_O solution. Cultures were maintained in 5% CO_2_ at 37°C, and half of the medium was changed at 1 day and 7 days after plating. Control conditions (astrocytes alone) were kept under similar tissue culture conditions as neuron-astrocyte co-cultures.

### COLD JET SEPARATION METHOD

For removal of neurons from the co-cultured astrocytes, we modified a method previously used for removal of Schwann cells from co-cultured fibroblasts (Jirsova et al., 1997). 12-well tissue culture plates were placed on ice and the medium was gently aspirated. The cells were carefully rinsed once with ice-cold phosphate buffered saline (PBS; pH 7.4). Next, using a 1 mL pipette tip, 1 mL of ice-cold PBS was pipetted onto the co-cultures using a concentric circular motion, ensuring the force of the stream contacted the entire well surface area, which was repeated three times. This resulted in specific detachment of neurons, which were aspirated, leaving purified astrocytes, which were harvested for molecular analysis. All together, the isolation of astrocytes from co-cultures for molecular isolation per well was performed within a timeframe of 30 s. When co-culture cold jet (CC-CJ) cultures were compared to astrocyte alone cultures, the later were also treated by cold jet, therefore called astrocyte-alone cold jet (AA-CJ) samples.

### IMMUNOCYTOCHEMISTRY

Astrocyte monolayers or astrocyte-neuron co-cultures, before or after cold jet, were fixed by 4% paraformaldehyde in 0.1 M PBS for 15 min at room temperature (RT). Cells were incubated 30 min in 0.1 M PBS containing 0.5% TritonX-100 and 10% normal goat serum, and subsequently incubated with appropriate primary antibody concentrations in incubating medium (0.1 M PBS, 0.1% TritonX-100, 2% normal goat serum) at 4°C overnight. Cells were washed three times for 10 min in 0.1 M PBS and incubated in incubating medium containing the appropriated secondary antibody for 2 h. Sections were washed two times with 0.1 M PBS, one time with water, and subsequently DNA counterstained in Hoechst reagent 33258 solution (Sigma). The primary antibodies were rabbit anti-GFAP (1:500; Dako) and chicken anti-microtubule-associated protein 2 (MAP2; 1:5000; Abcam). Secondary antibodies were donkey anti-rabbit Cy3 (1:400; Jackson laboratories), goat anti-chick Alexa 488 (1:400; Molecular probes).

### RNA ISOLATION, REVERSE TRANSCRIPTION, AND QUANTITATIVE PCR

Total RNA was extracted from 12-well plate and mRNA was isolated with Trizol Reagent (Invitrogen). RNA samples for microarray analysis were derived from two to four tissue culture wells, and microarray replicates were derived from different pools of animals, with five to eight animals per pool. An additional chloroform extraction was performed to remove traces of phenol and samples were suspended in H_2_O. The quality and quantity of RNA samples for microarray analysis were assessed on Agilent RNA chips with the Agilent 2100 Bioanalyzer (Agilent Technologies). For quantitative PCR, four samples were used that were different than those used for microarray analysis, and each sample was derived from different animals and four to six tissue culture wells. RNA integrity was determined using electrophoresis with RNA loading dye on a 1% agarose gel, RNA quantity was determined by photospectrometry (ND-1000; Thermo Scientific). Quantitative PCR reactions were done as described (Camargo et al., 2012).

### MICROARRAY ANALYSIS

Agilent whole rat genome 4x 44K chips were used. cDNA synthesis and labeling for microarray hybridization were performed according to the manufacturers’ instructions. Briefly, oligo dT primers were used and Cy3 and Cy5 labeling was done using the Agilent two-color RNA Spike-In Kit. 0.5 μg of Cy5 and Cy3-labeled cRNA targets were hydrolyzed and mixed for 30 min. Arrays were hybridized with Cy5 and Cy3 solution for 18 h at 60°C in a rotating hybridization chamber, using 1× hybridization solution (Agilent technologies). Subsequently, arrays were washed, spun dry and scanned using an Agilent scanner.

Analysis of the microarrays served two purposes: (1) detection of neuron-induced gene expression in astrocytes and (2) determination of the cold jet efficiency on the mRNA level. In total, four arrays were used. For the first purpose, two CC-CJ samples were labeled with Cy3 and two AA-CJ samples were labeled with Cy5. Each CC-CJ sample was hybridized together with an AA-CJ sample to an array. For checking the cold jet procedure on the mRNA level, two co-culture (CC) samples were labeled with Cy5 fluorescence and hybridized on an array together with a CC-CJ sample (Cy3). The microarray dataset can be found at National Center for Biotechnology Information Gene Expression Omnibus (NCBI GEO; accession number GSE52481).

Normalization of the raw data was performed, after feature extraction by Agilent software, by optimized local intensity-dependent normalization (OLIN) and optimized scaled local intensity-dependent normalization (OSLIN; Futschik and Crompton, 2005) using the R project for statistical computing (version 6.0; R project). Next, *M* values were scaled so that upregulated probes were associated with positive values and downregulated probes with negative values. The following criteria were used for selection of regulated transcripts: (1) for each array, the mean and standard deviation (SD) of the *M* values of all probes was calculated, and only mRNAs with *M* values equal or higher to the mean + 2× SD, or equal or lower to the mean - 2× SD were selected, (2) *M* values had to fulfill our criteria on both replicate arrays, (3) for mRNAs that were represented on the microarray with multiple probes, data were checked for consistency, and probes were only included in the analysis when replicate sequences gave comparable results, (4) mRNAs upregulated in the CC-CJ with AA-CJ comparison could represent left-over neuronal RNA when also strongly downregulated in the CC with CC-CJ comparison. Therefore, mRNAs upregulated in the CC-CJ with AA-CJ comparison where only included when downregulation in the CC with CC-CJ comparison was less than the mean - 2× SD. To assess consistency between technical and biological replicate samples, as well as between replicate probes, non-parametric correlations (using Spearman’s rho) within non-normalized red or green fluorescence samples were calculated. For technical/biological replicate samples, correlations were only performed on regulated probes as indicated by the cold jet-astrocyte alone arrays.

Thereafter, data were analyzed by TopGo (Alexa et al., 2006) using the R project for statistical computing. Overrepresented biological processes were identified by scoring statistical significance of predefined annotated groups based on the gene ontology (GO) database. Statistical significance of overrepresented predefined GO biological processes was scored using the Fisher exact test (Alexa et al., 2006).

## RESULTS

### EFFECTIVENESS OF THE COLD JET METHOD ON NEURON-ASTROCYTE CO-CULTURES

**FIGURE 1:**
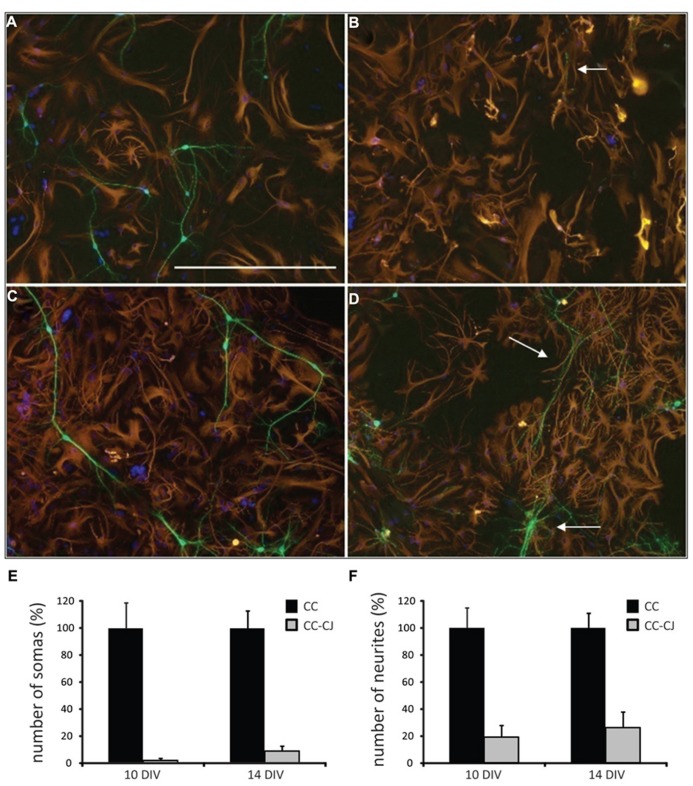
**Efficiency of cold jet on neuron-astrocyte co-cultures after 10 and 14 DIV.** **(A)** Neuron-astrocyte co-culture at 10 DIV. **(B)** At 10 DIV, cold jet removed almost all neuronal cell bodies and only a few residual neurites were detected (white arrow), note that the astrocyte cell layer remained unchanged. **(C) **Co-culture after 14 DIV. **(D)** At 14 DIV, cold jet removed neurites but less efficiently than at 10 DIV. Shown is an example were many residual neurites are observed (white arrows), probably representing clusters of firmly attached neurons. Neuron-astrocyte co-cultures with or without treatment with cold jet, were stained for the neuronal marker MAP2 (green), the astrocyte marker GFAP (red), and nuclei [4′,6-diamidino-2-phenylindole (DAPI), blue] to count the number of somas **(E)** and neurites **(F)**. Numbers show average and SEM for three tissue culture wells. Scale bar 100 μM **(A)**.

**FIGURE 2:**
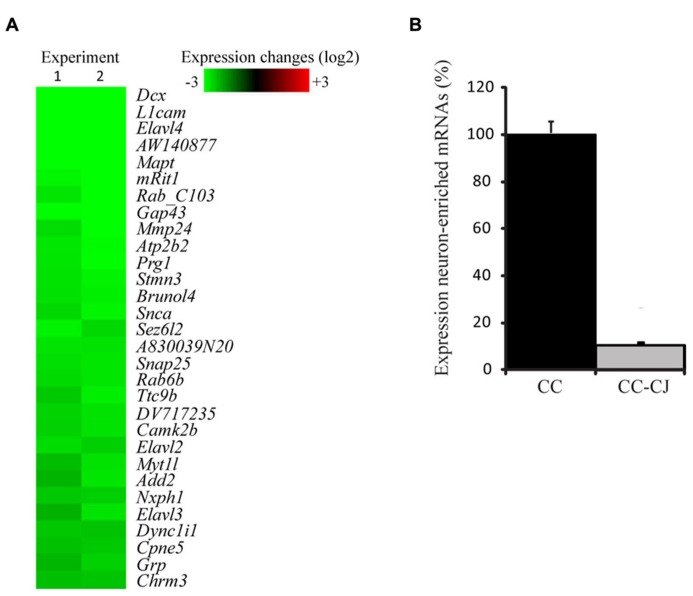
**Efficiency of cold jet to remove neuronal mRNA from co-cultures.** **(A)** Samples from neuron-astrocyte co-cultures, with or without treatment with cold jet, were collected and mRNA levels for neuronal mRNAs were analyzed using microarray. Shown are 40 of the most down-regulated transcripts in co-culture as a result of the cold jet procedure. Each individual gene expression level was normalized (see Methods) and plotted on a Log2 scale, green representing decreased mRNA levels in CC-CJ samples compared to CC alone **(B)** Normalized fluorescence levels were compared and expressed as percentage of the levels present in co-culture samples. Error bars represent standard error of the mean (SEM).

Neuronal development in hippocampal mixed neuron-glia cultures is a temporal process involving neurite outgrowth (1 DIV onward), and synapse formation from 4 DIV onward (Basarsky et al., 1994). Since attachment of neurons to the extracellular culture environment might vary with time, cold jet removal of neurons from neuron-astrocyte co-cultures was tested at 10 and 14 DIV. Quantification of the number of neuronal somas and neurites, indicated that cold jet removed over 98% of neuronal somas and 80% of neurites at 10 DIV, and was only slightly less efficient at 14 DIV (**Figure 1**). Cold jet removal of neuronal mRNAs was tested by microarray RNA profiling, which confirmed robust removal of neuronal mRNAs, e.g., *Mapt*, *Gap43*, *Snap25*, and *Snca* (**Figure 2A**). Neuron-enriched mRNAs are expected downregulated in coculture by cold jet, while being absent or low expressed in astrocytes. To determine the efficiency of the cold jet in removing these neuron-enriched mRNAs, their fluorescence levels in CC and CC-CJ was taken and the level in astrocytes substracted. The data indicated that the cold jet effectively removed approximately 90% of neuronal RNA (**Figure 2B**).

### GENE-EXPRESSION ANALYSIS OF NEURON-RESPONSIVE ASTROCYTE GENES

**FIGURE 3:**
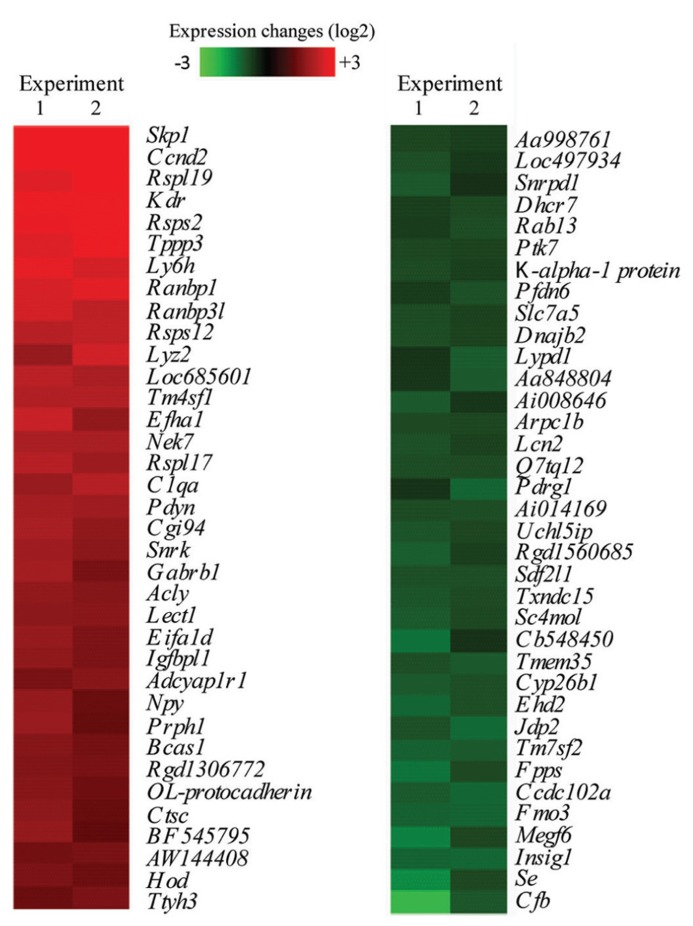
**Microarray analysis of strongest regulated neuron-responsive astrocyte genes.** Shown are 36 of the most up – and down-regulated transcripts in astrocytes when co-cultured with neurons, which were subsequent removed by cold jet treatment (CC-CJ), compared to astrocytes cultured alone (AA-CJ). Each individual gene expression level was normalized (see methods) and plotted on a Log2 scale with red representing increased and green decreased mRNA levels in CC-CJ astrocytes compared to astrocytes cultured alone (AA-CJ).

Microarray analysis of CC-CJ versus AA-CJ samples, yielded 394 neuron-induced regulations of astrocyte mRNAs, of which 270 were upregulated and 124 downregulated. The 36 strongest up- and down-regulated mRNAs are shown (**Figure 3**). The group of 270 mRNAs included many which are involved in astrocyte-neuron interactions, such as cell adhesion (*Itgb*, *Pcdh10*, *Cdh6*, *Ctnnal1*, *Glycam*, *L1cam,* and *TSP precursor 4*), extracellular matrix (*Cspg5* and *Reck*) and cytokine signaling (*Megf6 and Lcn2*). Several mRNAs involved in lipid synthesis were found down-regulated, including mRNAs involved in cholesterol synthesis (*Dhcr7*, *Hmgcs*, *Sc4mol*, *Cyp26b*, *Tm7sf2*, *FPS*, *Sql*, and *Insig1*).

To gain more insight into biological processes represented by the data set, we scored statistical significance of predefined functional genes groups, based on GO biological processes. From the complete list of regulated mRNAs (394), 10 significant overrepresented GO terms were identified (with *p* < 0.005, see **Table 1**). Overrepresented GO terms included mainly metabolic processes, e.g., sterol and nutrient related processes. GO analysis of only up-regulated (**Table 2**) or only down-regulated (**Table 3**) gene groups showed most strikingly that mRNAs associated with “sterol metabolism” were mainly down-regulated, whereas “response to nutrient” processes were up-regulated.

**Table 1:** Gene ontology-overrepresentation analysis for biological processes for all mRNAs (394) regulated in astrocytes when co-cultured with neurons.

GO ID	GO term	*p*-Value
GO:0016125	Sterol metabolic process	2.20E-10
GO:0006991	Response to sterol depletion	4.60E-07
GO:0031667	Response to nutrient levels	0.00019
GO:0045332	Phospholipid translocation	0.00049
GO:0015679	Plasma membrane copper ion transport	0.00082
GO:0000245	Spliceosome assembly	0.00089
GO:0001955	Blood vessel maturation	0.00288
GO:0007218	Neuropeptide signaling pathway	0.00291
GO:0006800	Oxygen and reactive oxygen species metabolism	0.00314
GO:0006968	Cellular defense response	0.00409

**Table 2:** Gene ontology-overrepresentation analysis for biological processes for mRNAs (270) up-regulated in astrocytes when co-cultured with neurons.

Gene ontology ID	Gene ontology term	*p*-Value
GO:0007584	Response to nutrient	0.00013
GO:0045332	Phospholipid translocation	0.00019
GO:0015679	Plasma membrane copper ion transport	0.00032
GO:0001955	Blood vessel maturation	0.00114
GO:0008406	Gonad development	0.00132
GO:0007218	Neuropeptide signaling pathway	0.00324
GO:0006911	Phagocytosis, engulfment	0.00326
GO:0007160	Cell-matrix adhesion	0.00475

**Table 3:** Gene ontology-overrepresentation analysis for biological processes for mRNAs (124) down-regulated in astrocytes when co-cultured with neurons.

Gene ontology ID	Gene ontology term	*p*-Value
GO:0016125	Sterol metabolic process	2.10E-11
GO:0006991	Response to sterol depletion	8.50E-09
GO:0000245	Spliceosome assembly	0.00055
GO:0006481	C-terminal protein methylation	0.00342
GO:0015819	Lysine transport	0.00342
GO:0046498	S-adenosylhomocysteine metabolic process	0.00382
GO:0006776	Vitamin A metabolic process	0.0047

**FIGURE 4:**
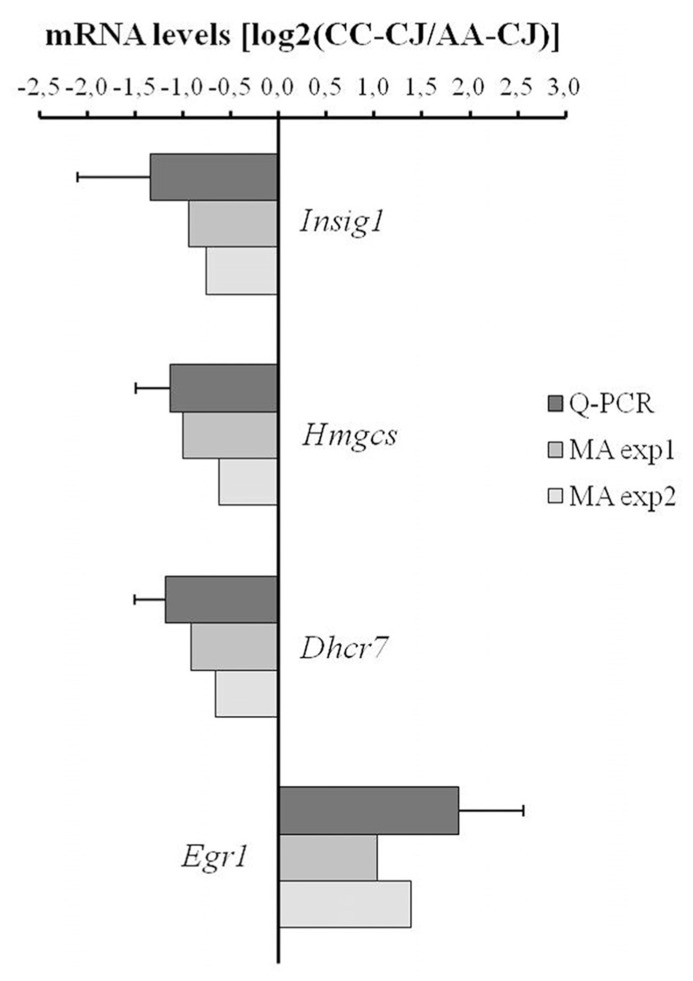
**Quantitative PCR analysis of neuron-responsive astrocytes genes.** Transcript levels were measured using qPCR for a selected group of astrocyte-expressed mRNAs that according to microarray were neuron-responsive (in MA exp 1 and MA exp 2). The ratio between expression in CC-CJ vs. AA-CJ was calculated and plotted on a Log2 scale, e.g., expression of EGR1 was 3.7-fold higher in CC-CJ then in AA-CJ. Data represents the mean and SD of four samples.

To validate the microarray data, qPCR was performed on a selection of mRNAs that were regulated between astrocytes alone (AA-CJ) and astrocytes in co-culture (CC-CJ). qPCR confirmed the down-regulation of three mRNAs involved in lipid metabolism (Insig1, Hmgcs, and Dhcr7) and confirmed the up-regulation of the transcription factor Egr1 in astrocytes when co-cultured with neurons (**Figure 4**).

## DISCUSSION

### COLD JET: METHOD FOR GENTLE SEPARATION OF NEURONS FROM CO-CULTURED ASTROCYTES

Neuron-glia co-cultures are an established *in vitro* model to investigate the reciprocal interactions of these cell types that underlie neurite outgrowth, synapse formation, and synaptic transmission. Investigating the molecular changes in astrocytes that take place during interaction requires the efficient isolation of astrocytes from the co-culture in a manner that ensures cellular integrity. Here, we present cold jet as a fast and efficient isolation method of astrocytes from differentiating neuron-astrocyte co-cultures. We showed that at 10 and 14 DIV, when neurites and mature synapses have been formed (Basarsky et al., 1994), cold jet removed nearly all neuronal RNA in these co-cultures. This permits selective transcriptional analysis of only astrocytes in these co-cultures. Cold jet isolation of astrocytes from neuron-astrocyte co-cultures may also be suitable for analysis of proteins and lipids, since more than 90% of neuronal cell bodies and nearly as much neurites are removed. We conclude that cold jet offers important advantages over other techniques that are used for cell separation, such as FACS or immuno-panning. Unlike those other techniques, cold jet is fast (within 1 min), easy to perform, done without addition of proteases, and conducted under ice-cold conditions to ensure no detriment to molecular integrity.

### IDENTIFICATION OF NEURON-RESPONSIVE ASTROCYTE GENES

Through a developmental *in vitro* model, in which neurons in co-culture with astrocytes differentiate and form synapses, we set out to identify neuron-responsive astrocyte gene expression using microarray analysis. We found regulation of many genes involved in astrocyte biological processes that have been reported previously to affect neuronal differentiation and function, such as lipid metabolism (Camargo et al., 2009, 2012; Pfrieger and Ungerer, 2011), cell-ECM adhesion (Christopherson et al., 2005; Tournell et al., 2006), complement factor signaling (Stevens et al., 2007), cytokine signaling (Acarin et al., 2001), and synaptic transmission (Halassa and Haydon, 2010). It should be noted that most of the astrocyte mRNAs involved in cholesterol metabolism were down-regulated by neuronal presence, which is surprising considering previous findings illustrating the importance of astrocyte cholesterol for synapse maturation and neurite outgrowth (Mauch et al., 2001; Fan et al., 2002; Hayashi et al., 2004; Camargo et al., 2009). However, we did find down-regulation of Insig1, an important negative regulator of the lipogenic sterol regulatory element binding protein (SREBP) transcription factors, which therefore would be expected to lead to increased SREBP-mediated transcription of lipogenic enzymes, as found in other cell systems (Engelking et al., 2005). Our recent observation that SREBPs are also required for expression of lipogenic enzymes in astrocytes (Camargo et al., 2012), warrants further investigation of the control of Insig, SREBP, and downstream transcription in astrocytes due to contact with neurons. Taken together, our observations suggest that astrocyte-factors involved in these processes do not only influence neuronal differentiation but are furthermore induced in astrocytes by neuronal presence, underscoring the reciprocal nature of the neuron-glia interactions.

## Conflict of Interest Statement

The authors declare that the research was conducted in the absence of any commercial or financial relationships that could be construed as a potential conflict of interest.
